# Integration of neural and epigenetic contributions to posttraumatic stress symptoms: The role of hippocampal volume and glucocorticoid receptor gene methylation

**DOI:** 10.1371/journal.pone.0192222

**Published:** 2018-02-07

**Authors:** M. Windy McNerney, Tong Sheng, Jordan M. Nechvatal, Alex G. Lee, David M. Lyons, Salil Soman, Chun-Ping Liao, Ruth O’Hara, Joachim Hallmayer, Joy Taylor, J. Wesson Ashford, Jerome Yesavage, Maheen M. Adamson

**Affiliations:** 1 Department of Psychiatry and Behavioral Sciences, Stanford School of Medicine, Stanford, California, United States of America; 2 War Related Illness and Injury Study Center (WRIISC), VA Palo Alto Health Care System, Palo Alto, California, United States of America; 3 Department of Radiology, Beth Israel Deaconess Medical Center, Harvard Medical School, Boston, Massachusetts, United States of America; 4 Mental Illness Research Education Clinical Centers (MIRECC), VA Palo Alto, Palo Alto, California, United States of America; 5 Defense and Veterans Brain Injury Center (DVBIC), VA Palo Alto, Palo Alto, California, United States of America; 6 Department of Neurosurgery, Stanford School of Medicine, Stanford, California, United States of America; The Ohio State University, UNITED STATES

## Abstract

Many Veterans exposed to physical and psychological trauma experience symptoms of posttraumatic stress disorder (PTSD). As the etiology of PTSD symptoms is complex, a better understanding of the underlying biological mechanisms may improve preventative care and treatment for PTSD. Recent findings from the fields of neuroimaging and epigenetics offer important insights into the potential brain structures and biochemical pathways of modified gene expression associated with PTSD. We combined neuroimaging and epigenetic measures to assess current PTSD symptoms by measuring overall hippocampal volume and methylation of the glucocorticoid receptor (GR) gene (promoter region). Multiple regression analyses indicated that the hippocampal volume/GR methylation interaction was a predictor of PTSD symptoms. Our findings suggest that neuroimaging and epigenetic measures contribute interactively to PTSD symptoms. Incorporation of these metrics may aid in the identification and treatment of PTSD patients.

## Introduction

Many Veterans exposed to physical and psychological trauma during combat continue to experience symptoms of posttraumatic stress disorder (PTSD) after service. Prevalence rates of PTSD are especially high among the Veteran population, at an estimated 15.2% of Vietnam Veterans approximately 10 years after the conclusion of the war, but the actual lifetime prevalence is thought to be much higher [1]. Within the Veteran Cohort, Gulf War, Operation Enduring Freedom/Operation Iraqi Freedom Veteran PTSD rates are estimated at 10.1% and 13.8%, respectively [2, 3].

The etiology of PTSD is complex and its confluence of symptoms with other mental health conditions make a clear diagnosis challenging. The symptoms of PTSD span across three distinct but correlated domains: 1) re-experiencing, 2) avoidance, 3) hyperarousal, and its high comorbidity with other disorders, especially depression and traumatic brain injury (TBI) [4] result in many Veterans being undiagnosed and untreated, exerting an enormous toll on the Veterans, their families, and society. Understanding complex psychiatric disorders such as PTSD requires research that integrates multiple scientific disciplines to objectively identify changes in PTSD.

Recent interest in epigenetic research may provide the tools to advance our understanding of the biochemistry behind PTSD. Animal research has shown that epigenetic modification of gene expression following environmental stress can influence stress-response functions, such as those mediated by the hypothalamic-pituitary-adrenal (HPA) axis [5, 6]. More recently, epigenetic factors, such as cytosine methylation, have been shown to modulate the effect of the environment on gene expression (6). Numerous studies have specifically focused on the exon 1F promoter region of the glucocorticoid receptor (GR) gene (*NR3C1*-1F), showing a considerable amount of variation related to psychiatric illnesses and early life stress, or possible environmental changes later in life (see [7, 8]). Hypomethylation of the *NR3C1-*1F region has also been found in the peripheral blood of Veterans with PTSD compared to Veterans without PTSD [9], which may explain changes in neuroendocrine functioning commonly observed with PTSD [10, 11].

*NR3C1*-1F cytosine methylation in patients with PTSD was recently found to be an important predictor of treatment response to psychotherapy, with higher methylation values associated with a positive response to treatment [12]. However, three months following treatment, there were no changes in methylation values in these participants, suggesting that peripheral methylation of the *NR3C1*-1F promoter may be an indicator of PTSD, rather than treatment progress. The research by Yehuda and colleagues has successfully laid the foundation for utilizing epigenetic cytosine methylation in PTSD research that could lead to effective diagnostic strategies [9,11,12]. Further investigations into the effects of the biochemical role of methylation in physiology will also provide useful information about the alterations in signaling pathways associated with PTSD.

It has recently been found that *NR3C1* methylation is positively correlated with hippocampal volume in patients with depression [13]. The hippocampus itself is sensitive to stress, and highly expresses the *NR3C1* gene. Taken together, these neuroimaging and epigenetic factors may provide the basis for an interesting avenue of research for PTSD symptomology. The relationship between hippocampal volume and PTSD is currently controversial in the literature. Imaging studies have found that Veterans with combat PTSD have a smaller hippocampal volume compared to Veterans without PTSD [14–17]. Others have failed to replicate this relationship [18, 19], or have focused on hippocampal size as a vulnerability to PTSD [20]. The likely reason for these inconsistent findings is that PTSD is very complicated, and understanding the biological underpinnings of this disorder requires more than just neuroimaging, but rather the integration of imaging and biochemistry information.

We therefore aimed to determine if the combination of brain imaging and epigenetics is a better predictor of PTSD symptomology than either factor alone by measuring hippocampal volume and cytosine methylation and utilizing this information in a complex regression model of PTSD symptom severity. We focus on the two canonical binding sites of the *NR3C1*-1F promoter containing 16 CpGs as these are the binding sites for nerve growth factor-induced clone A (NGF1A), which is important for regulating GR expression related to stress [21]. Although this is a small CpG area, we chose this particular region because it has previously been associated with PTSD [9, 12].

## Materials and methods

### Participants

The War-Related Illness & Injury Study Center (WRIISC) at the Palo Alto Veterans Affairs (VA) Medical Center is a part of a national effort to provide post-deployment care to Veterans with complex health problems. For eligibility and referral information, see http://www.warrelatedillness.va.gov/WARRELATEDILLNESS/referral/. Admitted patients typically have a complex medical history related to the chronic sequelae of TBI, mental health disorders (i.e. depression, PTSD), and other chronic conditions involving medical symptoms without a clear explanation. The high rates of TBI, mental health, chronic medical problems, and comorbidities within these WRIISC patients introduce an analytic challenge and obfuscate comparisons with studies involving individual Veteran cohorts (e.g., the Vietnam War: [22–24]; and OEF/OIF/OND conflict eras: [25, 26]). Although we recognize the complexity of our population, the interaction of PTSD with other disorders is beyond the scope of this study. Thus, in the current study we are primarily interested in PTSD symptoms, which are ubiquitous among our patients.

Data from Veteran patients (n = 67; age = 46.0 ± 10.8; 8 females) seen at the WRIISC at the Palo Alto VA were included in the current analyses. Prior to enrolling in the study, all participants gave informed consent. All Veteran patients were seen by providers at WRIISC and determined clinically to have capacity to consent. None of the patients enrolled in this study required Assent or a legally authorized representative and they provided their consent individually, after the study was explained to them by the study coordinator. The human subjects protocol was approved by the Institutional Review Board and Stanford University and the Department of Veterans Affairs, Palo Alto.

### PTSD symptom severity

Participants completed the PTSD Checklist (Civilian version 4; PCL-C), a self-report questionnaire that assesses global and domain-specific symptom severity over the last month. This is a 17-item measure that assess symptoms severity across three domains of PTSD (re-experiencing, avoidance, arousal). The sum of all the 17 responses (score range 17–85) is a global indicator of PTSD symptom severity. Scores can also be segmented to assess symptom severity across three domains, which were used to further investigate the contribution of hippocampal volume and methylation to these domains. The PCL-C has good test-retest reliability (>0.75), internal consistency (> 0.83), and a strong correlation with clinical standards [25, 27].

### Structural brain scan

Participants received neuroimaging under a clinical protocol using a GE Discovery MR750 3.0 T MRI scanner (G.E., Waukesha, WI, USA). The protocol included a volumetric 3D fast spoiled gradient echo (FSPGR) (TE Min Full; Flip angle 11 degrees; voxel size 1.2mm isotropic; TI 400; FOV 27 cm; NEX 1; Matrix 256x256).

#### Volume estimation

Cortical reconstruction and volumetric segmentation was performed with the Freesurfer 5.3 image analysis suite, which is documented and freely available for download (http://surfer.nmr.mgh.harvard.edu/). Specifically, total estimated intracranial volume and total hippocampal volume were produced. Final segmentations were reviewed by a neuroradiologist [28, 29].

### Biochemistry

Saliva samples via spit were also collected from participants using the collection kit from DNA Genotek. The cellular content of spit contains, in general, a higher ratio of leukocytes to epithelial cells than using buccal swabs. DNA was extracted from the samples using the DNA extraction kit from DNA Genotek (prepIT-L2P). The samples were mixed by inversion and incubated at 50°C for 2 hrs. A total of 500μL of saliva was mixed with 20μL of PT-L2P lysis buffer, incubated for 10min and centrifuged to remove impurities. The remaining supernatant was then added to 600μL of pure ethanol, mixed gently, and then centrifuged. The supernatant was removed and the pellet was washed with 250uL 70% ethanol. The DNA was eluted with 10mM Tris-Cl buffer containing 0.5mM EDTA, and allowed to rehydrate for at least 1hr with occasional vortexing. The DNA quantification and purity was checked by nanodrop.

#### Methylation quantification

**Fig 1:**
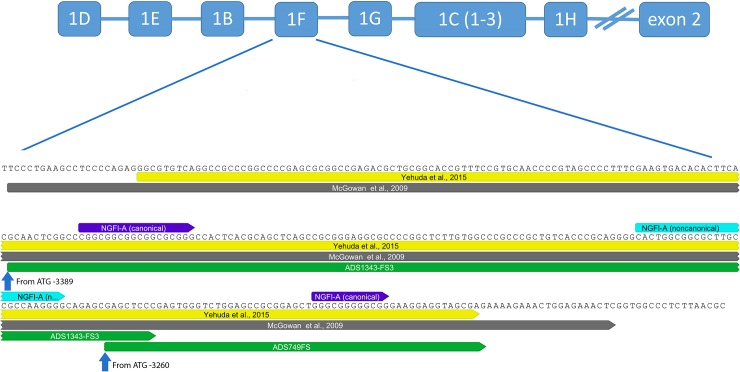
Map of the *NR3C1*-1F region. This was quantified for methylation in comparison to previous studies (8, 26), indicating canonical and noncanonical binding regions.

Methylation percent of the GR was carried out with pyrosequencing serviced by EpigenDx. Two assays, corresponding to ADS1343FS3 and ADS749FS were selected based on them being canonical NGFI-A binding sites and that they were within the GR region tested previously by Yehuda et al [9]; from the ATG start site, -3389 to -3252 and -3260 to -3201 respectively (Fig 1). Methylation is expressed as the percent of methylated to unmethylated CpG sites within the genome of interest. Due to the limited resources of clinical data, we were unable to analyze the samples in duplicate. Although we recognize this as a limitation in our study, our analysis was carried out by a commercial laboratory, which performed PCR bias testing (R^2^ = 0.98), quality control testing on the DNA, and has a high signal-to-noise ratio (21.4) with a documented low coefficient of variation (1.7) for assays on this region of interest, minimizing the likelihood of large variability from technical artifacts.

### Statistical analysis

First, the data was transformed prior to statistical modeling. Hippocampal volume was normalized to total intracranial volume, and age, hippocampal volume, PCL score, and GR methylation were all standardized to the standard deviation of the sample before analysis (Demographics shown in Table 1).

**Table 1:** Descriptive characteristics of the sample (n = 67).

Demographic	Mean	Standard Deviation
Age	46	10.9
Gender	8 females	…
Education	13.8	3.9
BMI	29.5	5.9
TBI History	65.70%	…
Blast TBI	4.30%	…
Deployed	19.10%	…
Combat Era		
OIF/OEF	36.90%	…
Desert Storm	46.20%	…
Vietnam	9.20%	…
Korea	3.10%	…
Other	13.90%	…
Multiple Eras	16.90%	…
GR Methylation	2.7	3
Norm hipp vol	5.2 x 10^−4^	8.5 x 10^−5^
PCL score	53	17.6

Abbreviations: traumatic brain injury (TBI); Operation Enduring Freedom and Operation Iraqi Freedom (OIF/OEF); glucocorticoid methylation (GR methyl); normalized (norm); hippocampus (hipp); volume (vol); PTSD Checklist (PCL). Hippocampal volume normalized to total intracranial volume.

To quantify specific associations between predictors and self-reported PTSD symptoms, we performed hierarchical regression modeling. Terms were entered into the regression in blocks, beginning with covariates (age, TBI history), main effects of predictors (methylation and hippocampal volume), and then the interaction effect between methylation and hippocampal volume to predict self-reported PTSD symptom severity. We did not include gender or type of TBI in the model because of the small numbers of females and blast-TBI in our sample, and preliminary data exploration revealed no systematic differences.

## Results

All hierarchical regression models were significant (R^2^s > 0.17; Ps < 0.002), largely due to the TBI covariate in the first two models. The main effects had little contribution relative to the covariate (Cohen’s *f* ^*2*^ = 0.01), while the interaction contributed a small/medium effect (Cohen’s *f* ^*2*^ = 0.11), and was a significant predictor (P = 0.024; see Table 2). The effect size measures for the model components were calculated in accordance with Soper [30] and Cohen [31]. To characterize the interaction term and the symptomology of PTSD, we segmented the data for secondary analyses as described below.

**Table 2:** Hierarchical regression model results. Standard coefficients are reported.

	Coefficeint (β)	Standard Error	ΔR^2^	Overall R^2^	*p*-value
Covariates			0.17	0.17*	0.002
Age	-0.08	0.11	…	…	0.616
TBI	0.77*	0.23	…	…	<0.001
Main Effect			0.01	0.18*	0.004
Hipp	-0.03	0.12	…	…	0.808
GR Meth	0.12	0.11	…	…	0.284
Interaction			0.08	0.26*	<0.001
Hipp x GR Meth	0.33*	0.14	…	…	0.024

Abbreviations: traumatic brain injury (TBI); methylation (GR Meth) hippocampus (Hipp)

* *p* < 0.05

### Secondary analyses

#### Interaction between methylation and hippocampal volume

**Fig 2:**
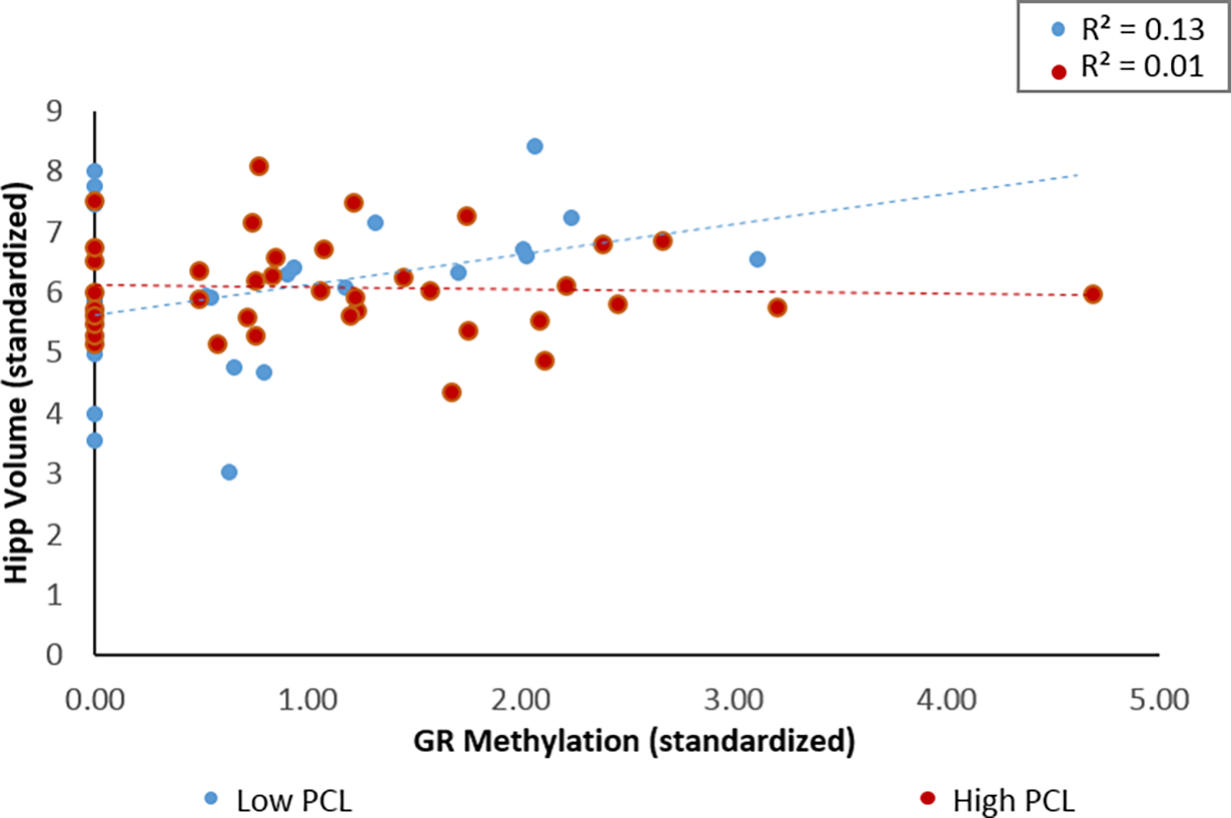
The relationship between GR methylation and hippocampal volumes for participants who are likely to have met diagnostic criteria for PTSD and those who likely do not. In patients who scored above the PCL-C cut-off of 50, no relationship was found, while a positive relationship was found for those who scored below.

To interpret the significant interaction between methylation and hippocampal volume, we ran correlation analyses between hippocampal volume and GR methylation on the participants that are likely to have met diagnostic criteria for PTSD (i.e., PCL-C > 50, *n* = 38) and those who likely do not (50 and below, *n* = 29) in accordance to previously published recommendations [32, 33] (see Fig 2). For those who scored low on the PCL-C, there was a positive correlation between methylation and hippocampal volume, which was marginally significant for the Pearson’s test (*r* = 0.36, *p* = .056) and significant for the Spearman test (*ρ* = .45, *p* = .016). The Pearson’s and Spearman’s correlations were nonsignificant for those with high PCL-C scores (Pearson’s: *r* = -0.04, *p* = .805; Spearman’s: *ρ* = .04, *p* = .818).

#### Hippocampus laterality

To observe the contributions of the left and right hippocampal volumes to self-reported PTSD symptom severity, we also tested hierarchical regression models that included either the left or right hippocampal volume instead of the combined hippocampal volume. We found that overall both the left and right hippocampus models were significant (left: *R*^2^ = 0.23, *p* = .002; right: *R*^2^ = 0.24, *p* = .001) and regression coefficients for the interaction terms (left: β = -0.28, SE = 0.13; right: β = -0.33, SE = 0.15) and effect sizes (Cohen’s *f* ^2^: left = 0.05, right = 0.06) were comparable between the two models. These results suggest that both left and right hippocampal volumes contributed similarly to self-reported PCL-C scores.

#### PTSD domains

The PCL-C assesses three domains of symptoms: re-experiencing, avoidance and hyperarousal. To focus discussion of the likely locus of PTSD symptoms on the interaction between methylation of hippocampal volume, we used three separate hierarchical models (Bonferroni corrected for multiple comparisons), with the three domains as the respective outcome variables (see Table 3). The overall models for re-experiencing (*R*^2^ = 0.30, *p* < .001) and arousal (*R*^2^ = 0.24, *p* < .01) were significant, while the avoidance model was marginally significant (*R*^2^ = 0.16, *p* < .06). The only significant interaction term was observed in the arousal model, which contributed a small/medium effect to the model (Cohen’s *f* ^*2*^ = 0.14), the interaction term other two models showed very small effects (re-experiencing: Cohen’s *f* ^*2*^ = 0.06; avoidance: Cohen’s *f* ^*2*^ = 0.05). These preliminary results indicate that the interaction between GR methylation and hippocampal volume could be an important marker for the arousal domain of PTSD.

**Table 3:** Assessing the model in predicting different domains of symptoms.

**Re-experiencing**	**Coefficient (β)**	**Standard Error**	**Overall R**^**2**^	***p*-value**
Covariates			0.23*	<0.001
Age	-0.05	0.12	…	0.908
TBI	0.87*	0.23	…	<0.001
Main Effect			0.25*	0.034
Hipp	-0.03	0.12	…	0.803
GR Meth	0.18	0.13	…	0.248
Interaction			0.30	0.343
Hipp x GR Meth	-0.27	0.14	…	0.182
** Avoidance**	**Coefficient (β)**	**Standard Error**	**Overall R**^**2**^	***p*-value**
Covariates			0.11	0.091
Age	-0.01	0.17	…	0.906
TBI	0.76*	0.32	…	0.027
Main Effect			0.12	0.487
Hipp	-0.08	0.17	…	0.641
GR Meth	0.21	0.18	…	0.439
Interaction			0.16	0.177
Hipp x GR Meth	-0.34	0.20	…	0.269
** Arousal**	**Coefficient (β)**	**Standard Error**	**Overall R**^**2**^	***p*-value**
Covariates			0.13*	0.034
Age	-0.10	0.12	…	0.211
TBI	0.60*	0.23	…	0.033
Main Effect			0.14	0.220
Hipp	-0.02	0.12	…	0.653
GR Meth	0.14	0.13	…	0.771
Interaction			0.24*	0.002
Hipp x GR Meth	-0.40*	0.14	…	0.015

The methylation x hippocampal volume interaction term was a significant predictor of arousal, but not of re-experiencing and avoidance. The Bonferroni correction for multiple comparisons was applied in these analyses. Abbreviations: traumatic brain injury (TBI); methylation (GR Meth) hippocampus (Hipp)

* p < 0.05.

## Discussion

Our findings suggest that neuroimaging and epigenetic measures contribute interactively to self-reported PTSD symptom severity. Our data revealed a significant interaction between *NR3C1* methylation and hippocampal size that was reflected in a positive relationship between methylation and hippocampal volume only for lower scores on the PCL-C. This effect was not specific to either hemisphere, and was strongest for the arousal domain of PTSD symptoms. These results indicate that more comprehensive research which incorporates measures from different sources can be particularly valuable in understanding PTSD pathology, and can provide valuable insights into the biochemical pathways of PTSD.

In the current set of data, splitting the samples by PCL-C score as a proxy for potential PTSD diagnosis revealed the loss of a relationship between *NR3C1*-1F methylation and hippocampal size. It has been well documented that there is a strong relationship between the hippocampus and the glucocorticoid stress response; the hippocampus can inhibit HPA axis activity, and hippocampal dysfunction can reduce inhibition of the HPA [34], potentially resulting in overexpression of stress signals. Glucocorticoid activity is damaging to hippocampal cells, through enhanced oxidative-stress [35], which, in turn, dysregulates glucocorticoid signaling. This would lead one to expect that lower methylation values would be associated with a smaller hippocampal size, which is what our Veterans who likely do not have PTSD are showing. However, because PTSD is associated with an overall hypomethylation, it appears that this relationship is lost, so that lower methylation values occur regardless of hippocampal size. Future research should be conducted to determine if this trend holds in healthy individuals, other cohorts of Veterans or civilians with PTSD, and even other psychiatric disorders.

Our exploratory analyses pointed to the arousal domain as the major outcome related to the interaction effect. This is aligned with the notion that stress and arousal directly influences HPA activity and glucocorticoid receptor function [36]. Previous research did not find a correlation between hyperarousal and methylation in patients with PTSD [37], but they also did not measure hippocampal volume, which is an important factor in the current data. This provides further rationalization to combine data in a multifaceted approach. An important next step would be to investigate if this pattern exists exclusively with PTSD or also in patients with other anxiety disorders, to determine the specificity of this finding and how chronic hyperarousal can influence GR signaling within the brain.

In this study, we focused on the *NR3C1*-1F promoter region because it is vital for the HPA stress response pathway [38], and has already been shown to be relevant to mental health, such as maternal depression [39], childhood trauma [40, 41], and PTSD [9]. The *NR3C1*-1F promoter is the human orthologue of the I_7_ region in the rat, and is comprised of 326 base pairs, containing 43 sites where a cytosine is adjacent to a guanine (CpG). Cytosine methylation commonly occurs on these CpG locations, resulting in methyl groups at a diagonal in the double helix configuration. Methylation occurs through the action of different forms of methyltransferase for *de novo* methylation, and maintenance of the methylation through DNA replication [40]. This is very stable, but demethylation can occur through oxidation by the ten-eleven translocation (TET) enzymes or DNA glycosylase [42]. Therefore, although cytosine methylation is generally stable, it can be dynamically altered throughout the lifespan, perhaps also in cases of trauma.

The effect of methylation on gene expression is complex, as it depends on the specific location where the methylation occurs [43]. It is generally thought that increased methylation on a promoter region downregulates gene expression through the impairment of transcription factor binding [44]. Therefore, hypomethylation is required for increased gene expression, which is what we found in our current data. Increased *NR3C1* promoter methylation has been shown to reduce basal transcriptional activity *in vitro* [45]. The same study also found hypermethylation of *NR3C1-*1F in the hippocampus of suicide and abuse victims along with decreased glucocorticoid receptor mRNA levels. Conversely, hypomethylation is typically associated with an increase in gene expression [46], so too little methylation can result in overexpression, which has been found in cases of PTSD (9). Therefore, there is a delicate balance with the amount methylation on a gene, which can provide a link between PTSD and increased sensitivity of the negative feedback component in the HPA axis [47].

In the brain, the hippocampus is an area rich in glucocorticoid receptors, which also expresses the *NR3C1*-1F promoter [7]. It has been documented that the hippocampus is sensitive to stress on a biochemical and structural level [48], and DNA methylation has been linked to changes in glucocorticoid receptor density in the hippocampus following exposure to environmental stress [49]. These findings suggest that chronic induced stress can manifest as methylation changes and eventual hippocampal volume loss [50], or that an innate hypomethylation state combined with a lower hippocampal volume can predispose individuals to PTSD [20]. It is currently unknown if PTSD is associated with a smaller hippocampal volume as some research have found a relationship [48], while others have not [49]. This controversy could be because not all of the important variables involved in PTSD and hippocampal volumes have been fully accounted for, such as DNA methylation. Further research should be conducted to elucidate this.

Our methylation analysis was done on saliva rather than blood or other tissue samples because saliva is a very noninvasive manner to collect DNA from participants that are likely hesitant to donate other samples. Although DNA methylation can vary with sample type, it has previously been shown that DNA extracted from whole saliva using our methods originates from blood leukocytes [51, 52]. Other studies on *NR3C1* methylation have shown similar results using brain and leukocytes [45, 53], and have argued for peripheral tissue samples as a suitable surrogate for brain methylation [7]. Unfortunately, we were unable to provide a cell count to verify the ratio of leukocytes to epithelial cells; but our vendor has assured us that the kit used provides a high ratio. In addition, DNA derived from blood is typically derived from many more cell types than saliva [54]. Although more research needs to be conducted on the association between peripheral and central DNA methylation, we are confident that our saliva samples provide results with relevant implications for the central nervous system in PTSD, especially given the significant interaction with hippocampal volume.

As noted above, only a very small CpG region was analyzed in the current study to target a specific critical region previously found to be hypomethylated with PTSD. Our main effect of methylation in this case was very small relative to the entire *NR3C1-*1F region, which was expected given that promoter regions typically have low methylation, and the nature of our Veteran population. This could have created a floor effect in our data, which should be examined further. To our surprise, the data in this study did not replicate prior findings that Veterans with PTSD had lower methylation values [9,11,12]. This could be due to several reasons, including differences in the clinical definition of PTSD (PCL-C score vs PTSD diagnosis), the lower overall methylation in our samples, and the fact that others extracted DNA from blood and we used saliva. Our sample size was also considerably smaller, which likely impacted our power to detect any significant main effects. Nonetheless, the interaction effect with hippocampal volume in this data was striking, as it is a novel finding and provides new information about the biochemistry of PTSD. These preliminary results pave the way to more research on potentially larger genomic regions or other brain areas.

Given the limitations of our clinical sample, we were unable to perform the pyrosequencing analysis in duplicate, which is more typically done in biochemistry. While we recognize this limitation, pyrosequencing has been shown to have low intraindividual variation among duplicates [55, 56], especially when carried out by a commercial laboratory. We believe it is likely that variability due to technical artifact had a little to no contribution to the results.

The current study did not include premorbid or longitudinal data, so we were unable to determine a causal relationship between PTSD symptoms, hypomethylation, and hippocampal volume. The PCL-C was administered to assess PTSD symptomology over the short-term (past month), so our findings related to methylation and hippocampal volume served as a potential indicator for current symptom severity. The PCL-C is a reliable self-report measure of PTSD that is administered to Veterans on a regular basis at the Palo Alto VA facility [27]. Another commonly used measure is the Clinician-Administered PTSD Scale (CAPS), which consists of 30-item structured interview. There is good correlation between the CAPS and PCL-C, with the PCL-C serving as a relevant brief screening tool for PTSD [25], so it is likely either metric is valid for this research.

Future research could be to investigate this relationship in terms of the different hippocampal subfields rather than focusing on gross anatomy. Glucocorticoid receptor expression is abundant in most subfields of the hippocampus [57], while PTSD may be particularly associated with smaller CA3/dentate gyrus volumes over other subfields [58]. We speculate it is possible that these subfields could play an important role in HPA signaling [59], and be a driving factor in the interaction found here. A recent investigation found a positive correlation between *NR3C1* methylation and cornu ammonis regions and dentate gyrus in patients suffering from depression that was absent in the control population [13], suggesting that methylation variability in psychiatric conditions could be associated with specific hippocampal subfields.

## Conclusions

The CA WRIISC Veterans have complex medical problems and high comorbidities, including high rates of PTSD, TBI and depression. This complicates comparison with other studies or populations. Also, our somewhat small sample size limited analytic capabilities beyond testing the main hypothesis, and the methylation biochemistry analysis was not done in duplicate. Even so, these preliminary results demonstrate that a multifaceted approach is necessary to fully investigate mental disorders in a complex patient population. Overall, these findings show a distinct pattern of results dependent on low vs high PTSD symptom severity, strengthening the idea that epigenetics and neural anatomy can be used as an effective indicator for PTSD.

## Supporting information

S1 Table PTSD methylation data. Raw data used in this research. Abbreviations: case identifier (ID); standardized PTSD CheckList (stPCL); PCL (PTSD CheckList); standardized methylation (StMethyl); traumatic brain injury (TBI), Standardized hippocampal volume (StHip); standardized education (StEdu); standardized right hippocampal volume (StRHip); standardized left hippocampal volume (StLHip); standardized re-experiencing (StReex); standardized avoidance (stAvoid); standardized arousal (StArous).
(TXT)
